# Enhancement of the HIV-1-Specific Immune Response Induced by an mRNA Vaccine through Boosting with a Poxvirus MVA Vector Expressing the Same Antigen

**DOI:** 10.3390/vaccines9090959

**Published:** 2021-08-27

**Authors:** Carmen Elena Gómez, Beatriz Perdiguero, Lorena Usero, Laura Marcos-Villar, Laia Miralles, Lorna Leal, Carlos Óscar S. Sorzano, Cristina Sánchez-Corzo, Montserrat Plana, Felipe García, Mariano Esteban

**Affiliations:** 1 Centro Nacional de Biotecnología (CNB), Department of Molecular and Cellular Biology, Consejo Superior de Investigaciones Científicas (CSIC), 28049 Madrid, Spain; perdigue@cnb.csic.es (B.P.); lmarcos@cnb.csic.es (L.M.-V.); cscorzo@cnb.csic.es (C.S.-C.); 2 AIDS Research Group, Institut d’Investigacions Biomèdiques August Pi i Sunyer (IDIBAPS), Hospital Clinic, University of Barcelona, 08036 Barcelona, Spain; usero@clinic.cat (L.U.); lmiralle@clinic.cat (L.M.); laleal@clinic.cat (L.L.); mplana@clinic.cat (M.P.); fgarcia@clinic.cat (F.G.); 3 Biocomputing Unit and Computational Genomics, CNB-CSIC, 28049 Madrid, Spain; coss@cnb.csic.es

**Keywords:** HIV-1 mRNA vaccines, multiepitopic protein, intranodal delivery, T cells, poxvirus MVA vector, mice, combined vaccines, prime/boost, immune responses

## Abstract

Development of a vaccine against HIV remains a major target goal in the field. The recent success of mRNA vaccines against the coronavirus SARS-CoV-2 is pointing out a new era of vaccine designs against pathogens. Here, we have generated two types of mRNA vaccine candidates against HIV-1; one based on unmodified vectors and the other on 1-methyl-3′-pseudouridylyl modified vectors expressing a T cell multiepitopic construct including protective conserved epitopes from HIV-1 Gag, Pol and Nef proteins (referred to as RNA-TMEP and RNA-TMEPmod, respectively) and defined their biological and immunological properties in cultured cells and in mice. In cultured cells, both mRNA vectors expressed the corresponding protein, with higher levels observed in the unmodified mRNA, leading to activated macrophages with differential induction of innate immune molecules. In mice, intranodal administration of the mRNAs induced the activation of specific T cell (CD4 and CD8) responses, and the levels were markedly enhanced after a booster immunization with the poxvirus vector MVA-TMEP expressing the same antigen. This immune activation was maintained even three months later. These findings revealed a potent combined immunization regimen able to enhance the HIV-1-specific immune responses induced by an mRNA vaccine that might be applicable to human vaccination programs with mRNA and MVA vectors.

## 1. Introduction

The human immunodeficiency virus (HIV), identified almost 40 years ago as the causative agent of acquired immunodeficiency syndrome (AIDS), continues to be lethal to humanity, accumulating more than 32 million deaths worldwide. Statistics reported by the World Health Organization (WHO) estimate that a total of 38 million people live with the infection worldwide. The introduction of antiretroviral therapy (ART) in the last century transformed the HIV infection from a death sentence to a manageable chronic disease. Globally there has been a reduction in morbidity and mortality related to HIV and currently a total of 25.4 million people have access to treatment, representing 67% of those infected. However, despite these great advances, about one million people die of HIV-related diseases and 1.7 million new infections occur worldwide each year (www.unaids.org, accessed on 1 August 2021).

Vaccines are one of the most cost-effective medical treatments in modern civilization, but the development of an effective vaccine against HIV/AIDS has proven to be one of the greatest complex scientific challenges. Seven HIV-1 vaccine efficacy trials have been completed, targeting both humoral and cellular immunity through distinct approaches. However, six of them failed and showed no protection. The only phase III clinical trial that has shown any degree of efficacy against HIV-1 infection was the RV144 trial, conducted in Thailand using more than 16,000 healthy heterosexual volunteers with moderate risk of HIV infection [1]. The results of this clinical trial revealed that the combination of a recombinant poxvirus based on the ALVAC strain with the monomeric HIV-1 Env protein using the “prime/boost” strategy provided 31.2% efficacy in preventing HIV-1 infection over the course of 42 months despite the absence of neutralizing antibodies (NAbs). For the first time, this trial provided evidence that an HIV/AIDS vaccine can prevent HIV-1 infection and highlighted that a heterologous regimen including poxvirus vectors should be considered in future HIV/AIDS vaccine strategies. Unfortunately, the phase III study, named HVTN 702, that used the same RV144 vaccine regimen adapted to the HIV-1 subtype clade C, which is the most common in Southern Africa, was ineffective in preventing HIV-1 infection and hence discontinued in early February 2020 (www.niaid.nih.gov, accessed on 1 August 2021). It is unclear whether this failure is related to the ethnic groups or to other factors. With this scenery, it is imperative to identify new target antigens and immunization strategies that are able to confer protection against infection to ensure “next-generation” vaccines.

As in natural infection, the immune responses elicited by vaccine candidates expressing full or near full-length HIV-1 natural proteins are biased towards non-beneficial targets masking the immune responses to the protective conserved ones. For this reason, epitope-based vaccines have emerged as an improved strategy to focus the immune responses towards selected epitopes. We have previously described the design and immunogenicity profile in the mouse model of a T cell HIV-1 immunogen containing different domains of HIV-1 Gag, Pol and Nef proteins, termed TMEP (T cell multiepitopic peptide) when expressed by DNA or MVA vectors [2,3]. Since mRNA vaccines represent a promising alternative to conventional vaccine approaches due to their high potency, rapid development, potential for low-cost manufacture and safe administration [4], here we evaluated in vitro and in vivo the potential of unmodified and modified mRNA vectors expressing the TMEP multiepitopic protein when used alone, or in prime/boost combination with an MVA vector expressing TMEP, to induce HIV-1-specific immune responses. Our findings highlight the benefits of priming with an mRNA vaccine followed by an MVA vector expressing the same antigen as a regimen to enhance the immune responses to HIV-1, a strategy that could be applied to other mRNA vaccines.

## 2. Materials and Methods

### 2.1. Cells and Viruses

The highly transfectable 293T cell line, derived from human epithelial embryonic kidney 293 cells containing the SV40 T-antigen, was grown in Dulbecco’s Modified Eagle’s Medium (DMEM) supplemented with 2 mM L-glutamine (Merck, Kenilworth, NJ, USA), 100 U/mL penicillin/100 µg/mL streptomycin (Sigma-Aldrich, St. Louis, MO, USA), 0.1 mM non-essential amino acids (Sigma-Aldrich), 0.5 μg/mL amphotericin B (fungizone; Gibco-Life Technologies, Waltham, MA, USA) and 10% heat-inactivated fetal calf serum (FCS; Sigma-Aldrich). The human THP-1 monocyte cell line was grown in Roswell Park Memorial Institute-1640 medium (RPMI-1640; Sigma-Aldrich) supplemented as above. THP-1 cells were differentiated into macrophages by incubation with 150 nM phorbol 12-myristate 13-acetate (PMA; Sigma-Aldrich) for 48 h. Cells were maintained in a humidified air 5% CO_2_ atmosphere at 37 °C.

The viruses used in this work included: the attenuated wild-type modified vaccinia virus Ankara (MVA-WT) obtained from the Ankara strain after 586 serial passages in CEF cells (provided by G. Sutter, Ludwig-Maximilians-University of Munich, Munich, Germany) and MVA-TMEP-B (shortly MVA-TMEP) in which the TMEP-B gene was inserted into the viral TK locus of the parental MVA-WT virus and previously described [3].

### 2.2. Blood Samples

Peripheral blood mononuclear cells (PBMCs) from healthy donors (HD) were isolated by Ficoll-Paque density-gradient centrifugation at 2000 rpm for 20 min at room temperature. The PBMCs collected were washed twice with phosphate-buffered saline 1X (PBS 1X) + 10% fetal bovine serum (FBS; Biowest, Riverside, MO, USA), counted for viability using trypan blue exclusion dye and used to obtain monocyte-derived dendritic cells (MDDCs).

### 2.3. Generation of MDDCs from HD

After isolation, PBMCs were immediately cultured in a T75 flask for 2 h at 37 °C in a humidified air 5% CO_2_ atmosphere at a concentration of 3–4 × 10^6^ cells/mL in X-VIVO 15 media (Lonza, Basel, Switzerland), supplemented with 1% human AB serum (Sigma-Aldrich), 50 μg/mL gentamicin (B/Braun Medical, Melsungen, Germany) and 2.5 μg/mL amphotericin B (fungizone; Bristol-MyersSquibb, Rueil-Malmaison, France). After 2 h of incubation, monocytes were observed as adherent cells and nonadherent cells were removed by three washes with PBS 1X. Subsequently, 1000 U/mL each of human recombinant IL-4 and GM-CSF (both from ProSpec, Rehovot, Israel) were added to monocytes (days 0 and 2) and after 5 days the immature MDDCs were obtained and washed with cold PBS 1X.

### 2.4. DNA and mRNA Vectors

DNA plasmids used in this work as priming agents for in vivo assays included pcDNA3.0 (shortly DNA-ϕ; Invitrogen, Carlsbad, CA, USA) and pcDNA-TMEP-B (shortly DNA-TMEP) previously described [2]. mRNA vectors used as priming agents included mRNA-TMEP and mRNA-TMEPmod (shortly RNA-TMEP and RNA-TMEPmod). Production of modified and unmodified TMEP mRNAs was carried out from the plasmid containing the TMEP multiepitope under the control of the T7 polymerase by a service contract with the company Trilink BioTechnologies, Inc. (San Diego, CA, USA).

### 2.5. TMEP Expression after mRNA Transfection of 293T, THP-1 and MDDCs by Flow Cytometry and Western Blotting

The expression of TMEP protein was determined by flow cytometry and western blotting, using a specific antibody against the FLAG tag located at the C-terminus of the TMEP sequence. For flow cytometry analysis, 1 × 10^6^ 293T cells were DNA-transfected (5 µg DNA-ϕ or DNA-TMEP) or mRNA-transfected (5 µg RNA-TMEP or RNA-TMEPmod) using Lipofectamine-2000 (Invitrogen) according to the manufacturer’s instructions. At 3, 6 and 16 h post-transfection, 2.5 × 10^5^ cells were collected for western blotting analysis (see below) and the rest of the transfected cells were harvested and filtered through a cell strainer using PBS 1X (Ca^−^/Mg^−^) to rinse the well, washed once with PBS 1X (Ca^−^/Mg^−^), resuspended in flow cytometry staining buffer (FACS buffer: PBS 1X (Ca^−^/Mg^−^)-2 mM EDTA–1% bovine serum albumin (BSA)) and seeded in a 96-well plate (200 µL/well). After centrifugation (5 min at 1500 rpm) and supernatant discard, cells were stained with the live/dead fixable red dye (1:200; Invitrogen) for 30 min at 4 °C in the dark, washed twice with FACS buffer and fixed/permeabilized with BD Cytofix/Cytoperm (BD Biosciences, San Jose, CA, USA) for 20 min at 4 °C. The cells were then centrifuged for 5 min at 1500 rpm, washed twice with PermWash (PW) 1X buffer (diluted in FACS buffer; BD Biosciences) and blocked with PBS 1X-3% BSA for 30 min at 4 °C. Next, the cells were incubated with 5 µg/mL of the monoclonal antibody anti-FLAG M2 (Sigma-Aldrich) in 50 μL PW 1X for 30 min at 4 °C in the dark. The cells were then washed twice with PW 1X and secondary anti-mouse IgG (H + L)-PE antibody (1:100; Beckman Coulter, Brea, CA, USA) in 50 μL PW 1X was added to the cells. After 30 min of incubation at 4 °C in the dark, the cells were washed twice with PW 1X, resuspended in FACS buffer and acquired in a FC500 1 Laser flow cytometer (Beckman Coulter) and data analyses were carried out using FlowJo software (Version 10.4.2; Tree Star, Ashland, OR, USA). Geometric mean fluorescence intensity (gMFI) values on the “live cells” gate were used to calculate the TMEP score by applying the formula: No. TMEP^+^ cells × gMFI/No. live cells.

For western blotting analysis, DNA- or mRNA-transfected 293T cells were harvested at 3, 6 and 16 h post-transfection (see above), centrifuged for 5 min at 3000 rpm, resuspended in Laemmli buffer with 2-mercaptoethanol (reducing conditions), fractionated by 8% SDS-PAGE and analyzed by western blotting using mouse monoclonal anti-FLAG M2 antibody (1:1000; Sigma-Aldrich) followed by goat anti-mouse-horseradish peroxidase (1:2000; Sigma-Aldrich) to evaluate TMEP expression. The immunocomplexes were detected by enhanced chemiluminescence system (ECL; GE Healthcare, Chicago, IL, USA). The same mRNA transfection assay was performed with human THP-1 cells at 3, 6 and 24 h post-transfection.

The MDDCs obtained from HD were mock-lipofected or lipofected with 15 µg of RNA (RNA-TMEP or RNA-TMEPmod) or 15 µg of DNA (DNA-ø or DNA-TMEP) using Lipofectamine-2000 (Invitrogen) according to the manufacturer’s instructions. Lipofected MDDCs were seeded in a 24-well plate at 5 × 10^5^ cells/well in the presence of 1000 U/mL each of rIL-4 and rGM-CSF (both from ProSpec) and 1000 U/mL of a maturation cytokine cocktail containing TNF-α, IL-6, IL1-β and PGE2 (CellGenix GmbH, Freiburg, Germany) was added. MDDCs were also mock-electroporated (w/o) or electroporated using 10 µg of RNA-TMEP or RNA-TMEPmod. Before electroporation, MDDCs were washed twice with Iscove’s Modified Dulbecco’s Medium (IMDM; Gibco, Waltham, MA, USA) without serum and centrifugated at 2000 rpm for 5 min. Then, 4 × 10^6^ MDDCs for each electroporation condition were resuspended in 400 µL of Ingenio^®^ Electroporation Kits and Solution (Mirus, Madison, WI, USA) following the manufacturer’s recommendations. The electroporation settings for both RNAs were the following: voltage of 300 V, capacitance of 150 vF and resistance of 800 Ω (rack 0.4 µm). After electroporation, the cells were transferred to fresh RPMI-1640 medium + 10% FBS and incubated for 3 h at 37 °C. After 3 h, MDDCs electroporated were seeded in a 96-well plate for 24 h using X-VIVO 15 media (Lonza), supplemented with 1% human AB serum (Sigma-Aldrich), 50 μg/mL gentamicin (B/Braun Medical) and 2.5 μg/mL amphotericin B (fungizone; Bristol-MyersSquibb) and in the presence of 1000 U/mL of rIL-4, rGM-CSF and maturation cytokine cocktail (TNF-α, IL-6, IL1-β and PGE2). At 6, 24 and 48 h post-transfection, the cells were collected for flow cytometry analysis to determine FLAG expression as mentioned above and maturation markers (see below).

Maturation markers on MDDCs such as CD86, CD80 and CD83 were analyzed by flow cytometry using the following fluorochrome-conjugated antibodies: anti-CD14-APC (Clone: M5E2) to analyze the percentage of monocyte contamination during the process of differentiation to MDDCs and CD86-FITC (Clone: 2331), CD80-PE (Clone: L307) and CD83-PE (Clone: HB15e) to determine MDDCs maturation level. All antibodies were purchased from BD Biosciences. Analyses were performed on CD14^−^ gated cells and relevant mouse immunoglobulin isotypes conjugated with PE, FITC or APC were used as controls for nonspecific binding. The viability of cell populations was assessed using the Annexin V-PE/7-AAD Apoptosis Kit (Becton Dickinson, Franklin Lakes, NJ, USA). Samples were acquired using a FACSCanto II (BD Biosciences) and data analyses were carried out using FlowJo software (Version 10.4.2; Tree Star).

### 2.6. RNA Analysis by Quantitative Reverse Transcription Real-Time PCR (RT-qPCR)

Total RNA from THP-1-transfected cells was isolated using the RNeasy Kit (Qiagen, Hilden, Germany). Reverse transcription using up to 1000 ng of RNA was performed with the QuantiTect reverse transcription kit (Qiagen), according to the manufacturer’s recommendations. qPCR was performed with a 7500 Real-Time PCR system (Applied Biosystems, Foster City, CA, USA) using Power SYBR green PCR Master Mix (Applied Biosystems). mRNA expression levels of RANTES, IFIT1, IFIT2, IFNβ, RIG-I, IL1β, IL6, IL10 and IL12 genes were analyzed by real-time PCR with specific oligonucleotides (sequences are available upon request). Specific gene expression was expressed relative to the expression of the cellular hypoxanthine phosphoribosyltransferase (HPRT) gene in arbitrary units (A.U.) using the 2−∆∆Ct method. All samples were tested in triplicate and two independent experiments were performed.

### 2.7. Peptides

The HIV-1 clade B consensus peptide pools used in this work included Gag-1 (55 peptides), Gag-2 (50 peptides), GPN-1 (53 peptides), GPN-2 (57 peptides), GPN-3 (56 peptides) and GPN-4 (55 peptides). They were provided by the National Institutes of Health (NIH) AIDS Research and Reference Reagent Program (Bethesda, MD, USA) and covered the HIV-1 Gag, Pol and Nef antigens from clade B as consecutive 15-mers overlapping by 11 amino acids. The pools that span the different fragments included in the TMEP construct have been previously described [2]. To analyze the HIV-1-specific cellular immune responses, we combined the different peptide pools as follows: Gag pool (Gag-1 + Gag-2) and GPN pool (GPN-1 + GPN-2 + GPN-3 + GPN-4). Vaccinia virus (VACV) E3_140–148_ peptide (sequence: VGPSNSPTF; CNB-CSIC Proteomics Service, Madrid, Spain), previously described as an immunodominant epitope in BALB/c mice [5], was used to determine VACV-specific CD8 T cell responses.

### 2.8. Ethics Statement

Blood was collected at the Banc de Sang i Teixits (BST) of Barcelona from 3 volunteer blood donors. This study received the approval of the Committee of Ethics and Clinical Investigation of the Hospital Clinic Universitari (Barcelona, Spain). Heparinized blood samples were collected after signing a written informed consent approved by the ethical committee of the relevant institutions.

Animal experimental protocols were approved by the Ethical Committee of Animal Experimentation (CEEA) of Centro Nacional de Biotecnología (CNB-CSIC, Madrid, Spain) according to Spanish National Royal Decree RD 53/2013, Spanish National Law 32/2007 on animal welfare, exploitation, transport and sacrifice and International EU Guidelines 2010/63/UE on protection of animals used for experimentation and other scientific purposes (permit number PROEX 281/16).

### 2.9. Mouse Immunization

Female BALB/c mice (6–8 weeks old) were purchased from ENVIGO (Gannat, France). For the first in vivo study, groups of BALB/c mice (*n* = 4) received three priming immunizations at days 0, 7 and 14 with 10 µg RNA-TMEP (groups 1 and 2), 10 µg DNA-TMEP (group 6) or PBS (groups 3, 4 and 5) into the inguinal lymph node (LN) (10 µL: intranodal route). Ten days later (day 24), animals from groups 1 and 3 were sacrificed and their spleens and inguinal LNs processed for intracellular cytokine staining (ICS) assay to determine the HIV-1-specific cellular immune responses. The remaining groups (2, 4, 5 and 6) were immunized one month after the third intranodal immunization (day 44) with 1 × 10^7^ pfu of MVA-WT (group 5) or 1 × 10^7^ pfu of MVA-TMEP (groups 2, 4 and 6) (100 µL: intramuscular route). At 10 days post-MVA boost (day 54), the animals were sacrificed and their spleens and inguinal LNs processed for ICS assay to determine both HIV-1- and VACV-specific cellular immune responses. 

For the second in vivo study, groups of BALB/c mice (*n* = 4) received three priming immunizations at days 0, 7 and 14 with 10 µg RNA-TMEP (groups 1 and 2), 10 µg RNA-TMEPmod (groups 3 and 4) or PBS (groups 5, 6 and 7) into the inguinal LN (10 µL: intranodal route). Ten days later (day 24), animals from groups 1, 3 and 5 were sacrificed and their spleens and inguinal LNs processed for ICS assay to determine the HIV-1-specific cellular immune responses. The remaining groups (2, 4, 6 and 7) were immunized three and a half months after the third intranodal immunization (day 113) with 1 × 10^7^ pfu of MVA-WT (group 7) or 1 × 10^7^ pfu of MVA-TMEP (groups 2, 4 and 6) (100 µL: intramuscular route). At 10 days post-MVA boost (day 123), the animals were sacrificed and their spleens and inguinal LNs processed for ICS assay to determine both HIV-1- and VACV-specific cellular immune responses. 

### 2.10. Analysis of the TMEP-Specific CD4 and CD8 T Cell Responses by ICS Assay

To analyze the magnitude and phenotype of the HIV-1- and VACV-specific CD4 and CD8 T cell responses at the different time points analyzed (days 24, 54 and 123), 2 × 10^6^ splenocytes or lymphocytes from inguinal LNs (erythrocyte-depleted) were seeded on 96-well plates and stimulated ex vivo for 6 h in complete RPMI-1640 medium with 10% FCS, 1 μL/mL Golgiplug (BD Biosciences), anti-CD107a-FITC (BD Biosciences) and 5 μg/mL of the HIV-1 clade B consensus peptide pools or 10 μg/mL of the VACV E3 peptide (E3 peptide was only added for the analysis at days 54 or 123). Non-stimulated samples (RPMI) were used as control. After stimulation, cells were washed, stained for surface markers, permeabilized and stained intracellularly. Dead cells were excluded using the violet LIVE/DEAD stain kit (Invitrogen). For the analysis of CD4 and CD8 T cell immune responses, the following fluorochrome-conjugated antibodies were used: IFN-γ-PE-Cy7, IL-2-APC and TNF-α-PE for functional analyses and CD3-PE-CF594, CD4-APC-Cy7, CD8-V500, CD19-SPRD, Gr-1-SPRD and CD49b-Alexa 700 for phenotypic analyses. All antibodies were from BD Biosciences. Cells were acquired in a GALLIOS flow cytometer (Beckman Coulter), and the analysis of the data was performed using FlowJo software (Version 10.4.2; Tree Star). The number of lymphocyte-gated events ranged between 10^5^ and 5 × 10^5^.

### 2.11. Data Analysis and Statistics

For the analysis of flow cytometry data from 293T and THP-1 transfected cells as well as for RT-qPCR data, a one way ANOVA test followed by Tukey’s honest significant difference criterion was performed. Analyses of flow cytometry data from transfected MDDCs were performed using parametric (Student’s *t*-test) or nonparametric (Mann–Whitney or Wilcoxon signed rank test) tests as appropriate. Statistical analysis was performed using GraphPad Software (GraphPad Prism version 5.00; La Jolla, CA, USA). For the analysis of ICS data, a statistical approach that adjusts the values for the non-stimulated controls (RPMI) and calculates the confidence intervals and *p* values was used [6]. Only antigen responses significantly higher than the corresponding RPMI samples are represented. All the values indicated are background subtracted.

## 3. Results

### 3.1. Differential Expression of Unmodified versus Modified mRNAs and Comparison with a DNA Vector in Cultured Cells

**Figure 1:**
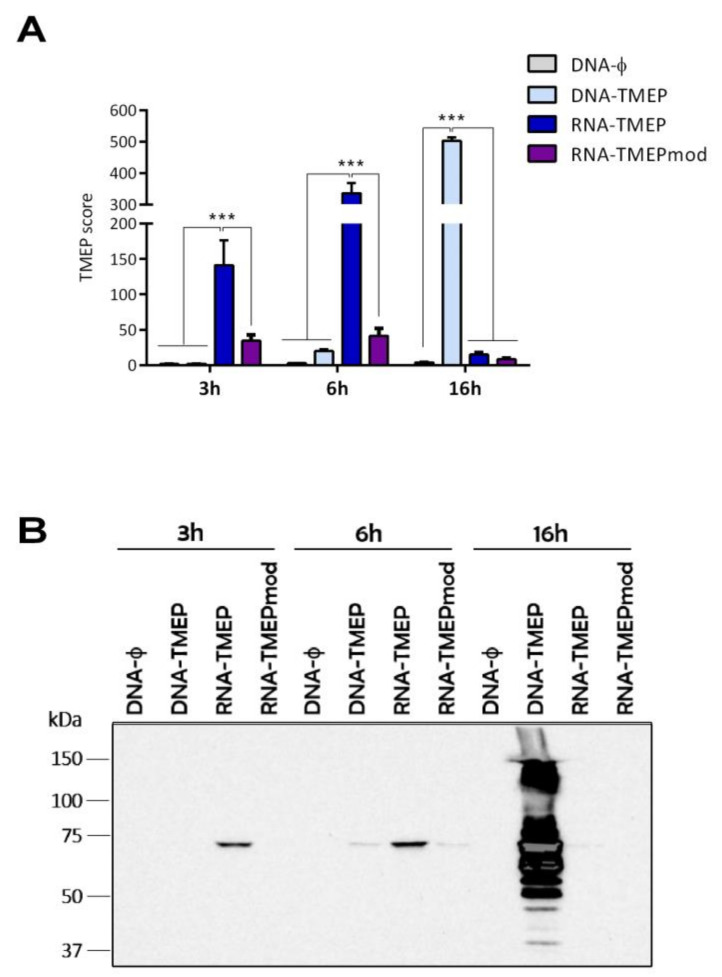
TMEP expression analysis after mRNA transfection of 293T cells by flow cytometry and western blotting using a specific antibody against the FLAG tag. 293T cells were DNA-transfected (5 µg DNA-ϕ or DNA-TMEP) or RNA-transfected (5 µg RNA-TMEP or RNA-TMEPmod) and at 3, 6 and 16 h post-transfection cells were collected for flow cytometry (**A**) or western blotting analyses (**B**). (**A**) For flow cytometry, cells were processed as described in Materials and Methods using 5 µg/mL of the monoclonal antibody anti-FLAG M2. Geometric mean fluorescence intensity (gMFI) values on the “live cells” gate were used to calculate the TMEP score by applying the formula: No. TMEP^+^ cells × gMFI/No. live cells. *** *p* < 0.001. (**B**) For western blotting analysis, DNA- or RNA-transfected 293T cells were harvested at 3, 6 and 16 h post-transfection, fractionated by 8% SDS-PAGE and TMEP expression analyzed by western blotting using the mouse monoclonal anti-FLAG M2 antibody.

The kinetic of expression of TMEP protein from 293T cells transfected with either DNA-TMEP or RNA-TMEP vectors was determined in permeabilized cells by flow cytometry using a specific antibody against the FLAG tag incorporated at the C-terminus of the TMEP construct. As shown in Figure 1A, a clear difference in the kinetics of TMEP expression between DNA- and RNA-transfected cells was observed. In RNA-transfected cells, TMEP expression was detected as early as 3 h post-transfection, peaking at 6 h and drastically declining at 16 h while in DNA-transfected cells TMEP protein was first detected at 6 h post-transfection at very low levels, peaking at 16 h. Earlier detection of TMEP protein in RNA-transfected cells is due to the mRNA being directly translated once it reaches the cytoplasm of the target cells whereas DNA has to enter the nucleus to be transcribed and processed thereafter. Levels of TMEP expression from unmodified RNA-TMEP-transfected cells were higher than the levels observed in the RNA-TMEPmod-transfected cells at 3 and 6 h post-transfection (*p* < 0.001). The above observations were also confirmed by western blotting analysis (Figure 1B). These results indicate an efficient expression of TMEP protein from cells transfected with DNA or mRNA vectors.

### 3.2. Time-Course Expression of TMEP Protein from MDDCs Transfected with RNA (Unmodified or Modified) or DNA Vectors

To determine TMEP protein expression in MDDCs transfected with RNA or DNA vectors, MDDCs were transfected with RNA-TMEP, RNA-TMEPmod or DNA-TMEP using two different transfection methods, electroporation and lipofection. TMEP expression at different time-points (6, 24 and 48 h post-transfection) was determined by flow cytometry using a specific antibody against FLAG tag.

**Figure 2:**
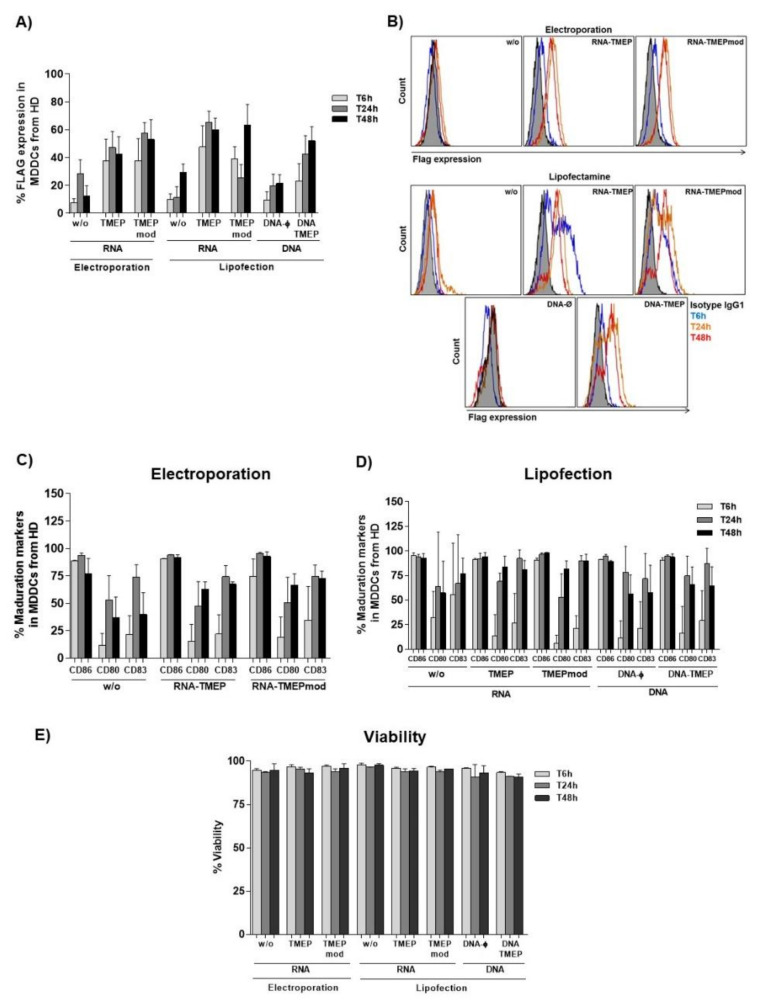
TMEP expression, DC maturation and cell viability analyses after RNA or DNA transfection of MDDCs from HD by flow cytometry. MDDCs were mock-transfected (w/o) or transfected with 10 µg (electroporation) or 15 µg (lipofection) of RNA-TMEP, RNA-TMEPmod or DNA-TMEP. MDDCs lipofected with 15 µg of DNA-ø were used as a control. At 6, 24 and 48 h post-transfection, cells were collected and processed for flow cytometry analysis as described in Materials and Methods. (**A**,**B**) FLAG expression was analyzed using 5 µg/mL of the monoclonal antibody anti-FLAG M2. (**C**,**D**) DC maturation of MDDCs electroporated (**C**) or lipofected (**D**) was analyzed using specific antibodies for CD86, CD80 and CD83 markers. (**E**) Cell viability of MDDCs was determined using Annexin V-PE/7-AAD.

In RNA or DNA-electroporated MDDCs, TMEP expression was detected at 6 h post-transfection, peaking at 24 h and declining at 48 h with a trend towards higher protein expression levels in RNA-TMEPmod-electroporated MDDCs than in RNA-TMEP-electroporated cells (Figure 2A,B). In MDDCs lipofected with RNA vectors, TMEP expression levels were slightly higher than the levels obtained using electroporation, but the kinetic expression profile was similar. However, when TMEP expression was analyzed in MDDCs lipofected with RNA-TMEPmod, the lowest expression level was observed at 24 h post-transfection (Figure 2A). For MDDCs lipofected with DNA-TMEP, the highest TMEP expression level was observed at 48 h post-transfection (Figure 2A). As expected, protein expression from RNA-TMEP vectors occurred earlier than that from DNA-TMEP. No statistically significant differences were observed at any analyzed time point.

### 3.3. Maturation of MDDCs Induced by RNA or DNA Vectors Expressing TMEP Multiepitopic Protein

We next analyzed the ability of RNA or DNA vectors expressing TMEP multiepitopic protein to induce the maturation of MDDCs. For this, the expression of different maturation markers, such as CD86, CD80 and CD83, was analyzed in MDDCs electroporated or lipofected with RNA-TMEP, RNA-TMEPmod or DNA-TMEP at 6, 24 and 48 h post-transfection by flow cytometry (Figure 2C,D). The highest expression levels were observed for CD86 marker, and these expression levels were similar between electroporated and lipofected MDDCs and using RNA- or DNA-based vectors and were maintained at the different analyzed time points (Figure 2C,D). In addition, CD80 and CD83 expression levels were lower than those obtained for CD86 marker regardless of whether MDDCs had been electroporated or lipofected. When MDDCs were electroporated with RNA-TMEP or RNA-TMEPmod the expression level of CD80 was slightly higher at 48 h than at 24 h while CD83 expression levels were similar at both 24 h and 48 h (Figure 2C). The same results were obtained when MDDCs were lipofected with RNA-TMEP or RNA-TMEPmod (Figure 2D). In contrast, when MDDCs were lipofected with DNA-TMEP, CD80 and CD83 expression levels were slightly higher at 24 h than at 48 h (Figure 2D). Similar results were obtained when negative controls were analyzed. Data were not statistically significant in any case.

Neither electroporation nor lipofection of MDDCs with RNA or DNA vectors affected cell viability since at all analyzed time points approximately 90–95% of MDDCs were alive (Figure 2E).

### 3.4. Expression of Unmodified and Modified RNAs in THP-1 Cells Differentially Affects the Levels of Proinflammatory Cytokines and Chemokines

**Figure 3:**
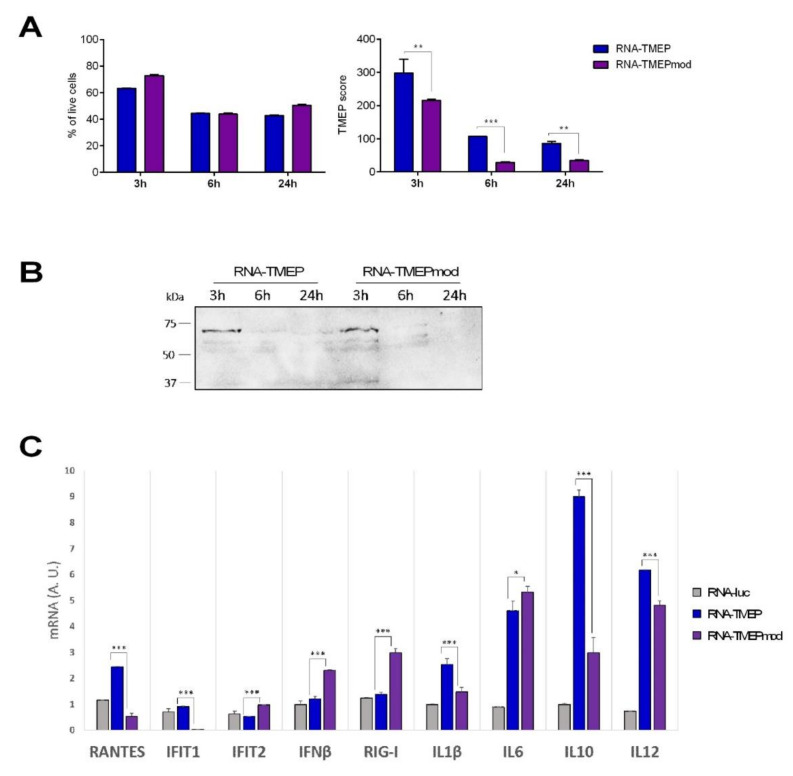
Effect of RNA-TMEP transfection on human THP-1 cells. (**A**,**B**) THP-1 macrophages were transfected with RNA-TMEP or RNA-TMEPmod and at 3, 6 or 24 h post-transfection TMEP expression was analyzed by flow cytometry (**A**) or western blotting (**B**). In both cases, the detection was performed using a specific anti-FLAG antibody. Geometric mean fluorescence intensity (gMFI) values on the “live cells” gate were used to calculate the TMEP score by applying the formula: No. TMEP^+^ cells × gMFI/No. live cells. ** *p* < 0.01; *** *p* < 0.001. (**C**) Proinflammatory cytokine and chemokine profile induced at 6 h post-transfection by RT-qPCR in THP-1 cells transfected with RNA-TMEP, RNA-TMEPmod or RNA-luc (used as control). Results are expressed as the ratio of the gene of interest to the control previously normalized by HPRT mRNA levels. A.U.: arbitrary units. * *p* < 0.05; *** *p* < 0.001. Means and standard deviations of triplicate samples from one experiment are represented. Data shown are representative of two independent experiments performed.

We next analyzed the potential effect of the mRNA modification in the context of a cell line with APC (antigen-presenting cell) features. For this, the kinetics of cell viability and expression of TMEP protein from THP-1 cells differentiated into macrophages and transfected with either RNA-TMEP or RNA-TMEPmod vectors were determined by flow cytometry in permeabilized cells by cell count or using a specific antibody against the FLAG tag, respectively. As shown in Figure 3A (left panel), there was no difference in cell viability after transfection with the two mRNAs. TMEP expression level was higher in unmodified RNA-TMEP-transfected cells than in cells transfected with the modified RNA version in the three time points analyzed. With both vectors the highest expression level was observed at 3 h post-transfection, declining thereafter (Figure 3A, right panel). The same expression pattern and kinetic profiles were confirmed by western blotting (Figure 3B).

For the analysis of the proinflammatory profile induced by RNA-TMEP transfection, human THP-1 macrophages were transfected with RNA-TMEP, RNA-TMEPmod or RNA-luc (used as a negative control). At 3 and 6 h post-transfection, RNA was extracted and RANTES, IFIT1, IFIT2, IFNβ, RIG-I, IL1β, IL6, IL10, IL12 and HPRT mRNA expression levels were analyzed by RT-qPCR. As shown in Figure 3C, mRNA expression levels of RANTES, IFIT1, IL1β, IL10 and IL12 in RNA-TMEP-transfected cells were higher than the levels observed in RNA-TMEPmod-transfected cells, while the levels of IFIT2, IFNβ, RIG-I and IL6 were increased in RNA-TMEPmod-transfected cells compared to RNA-TMEP transfection at 6 h. At 3 h post-transfection the results obtained were similar and luciferase expression was confirmed at both time points (data not shown). The above observations indicate a differential induction of immunomodulatory molecules by the two mRNAs. Such induction could play a role in the host innate response.

### 3.5. The Cellular Immune Response Induced in Mice by Unmodified RNA-TMEP Is Markedly Enhanced When Combined with the Poxvirus Vector MVA

**Figure 4:**
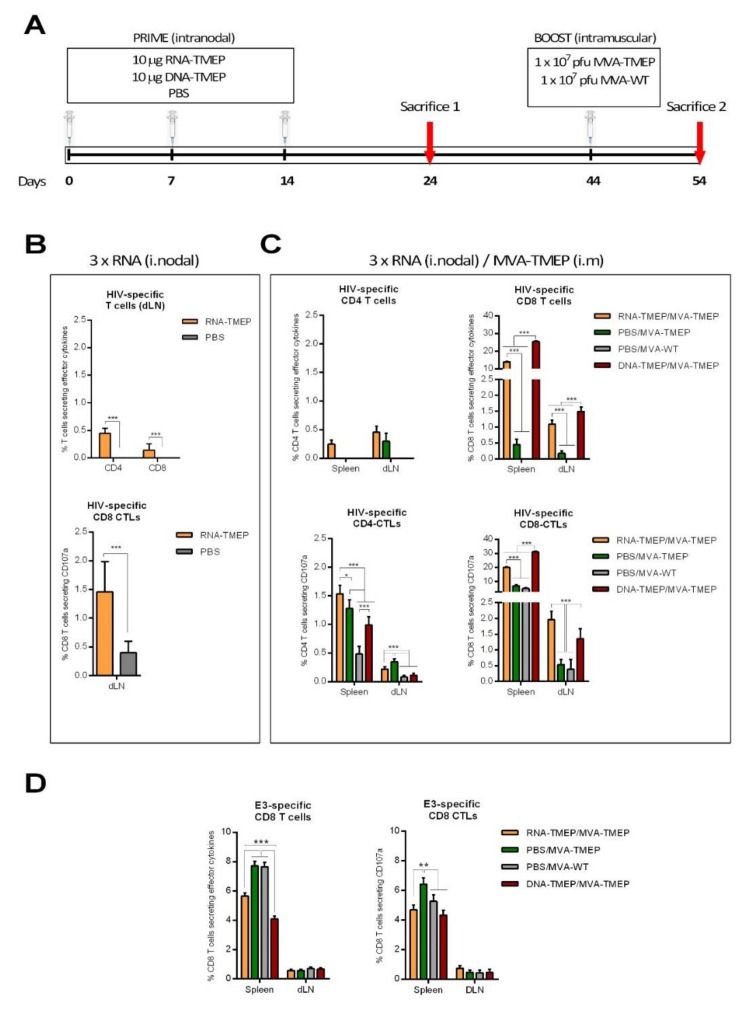
HIV-1- and VACV-specific T cell responses elicited in spleen and inguinal LNs after intranodal RNA-TMEP prime followed by intramuscular MVA-TMEP boost. (**A**) Immunization schedule. (**B**) Magnitude of the total HIV-1-specific CD4 and CD8 T cell responses measured at 10 days after the third intranodal immunization by ICS assay following stimulation of lymphocytes from inguinal LNs with HIV-1 clade B consensus peptide pools. Upper panel: The total value in each group represents the sum of the percentages of CD4^+^ or CD8^+^ T cells secreting IL-2 and/or IFN-γ and/or TNF-α effector cytokines. Lower panel: The total value in each group represents the percentages of CD8^+^ T cells expressing CD107a against HIV-1 clade B consensus peptide pools. (**C**) Magnitude of the TMEP-specific CD4 and CD8 T cell responses measured after intramuscular MVA boost by ICS assay following stimulation of splenocytes or lymphocytes from inguinal LNs with HIV-1 clade B consensus peptide pools. The total value in each group represents the sum of the percentages of CD4^+^ or CD8^+^ T cells secreting IL-2 and/or IFN-γ and/or TNF-α effector cytokines (upper panels) or the percentages of CD4^+^ or CD8^+^ T cells expressing CD107a (lower panels) against HIV-1 clade B consensus peptide pools. (**D**) Magnitude of the VACV E3-specific CD8 T cell responses measured after intramuscular MVA boost by ICS assay following stimulation of splenocytes or lymphocytes from inguinal LNs with VACV E3 peptide. The total value in each group represents the sum of the percentages of CD8^+^ T cells secreting IL-2 and/or IFN-γ and/or TNF-α (left panel) or the percentages of CD8^+^ T cells expressing CD107a (right panel) against E3 peptide. All data are background subtracted. 95% CI is represented. * *p* < 0.05; ** *p* < 0.005; *** *p* < 0.001.

Once we verified the correct expression of TMEP protein from DNA and mRNA vectors in transfected cells, we next defined the immune behavior of the best-in-class unmodified mRNA in a mouse model and the effect of homologous versus heterologous prime/boost combinations of vectors. To this aim we characterized in vivo the T cell immune responses induced by intranodal administration of RNA-TMEP alone or in combination with a viral vector MVA-TMEP administered as a booster. For comparative purposes, we also included in the analysis the heterologous combination of prime/boost with DNA-TMEP/MVA-TMEP. For this, groups of BALB/c mice were immunized as described in the Materials and Methods section. At 10 days post-MVA boost (day 54), the animals were sacrificed and their spleens and inguinal LNs processed for ICS assay to determine both HIV-1- and VACV-specific T cell responses (Figure 4A).

To analyze the HIV-1-specific T cells, splenocytes or lymphocytes from inguinal LNs were stimulated ex vivo for 6 h with a panel of 326 peptides from HIV-1 clade B consensus, spanning the different Gag, Pol and Nef fragments included in the TMEP construct. Vector-specific responses after MVA boost were detected using the VACV E3_140–148_ peptide. After stimulation, cells were incubated with specific antibodies to identify T cell lineage (CD3, CD4 and CD8), effector cytokines (IL-2, IFN-γ and TNF-α) and degranulation (CD107a) to define responding cells. The HIV-1- or VACV-specific T cell responses were established by the percentage of T cells with CD4 or CD8 phenotype that produced IL-2 and/or IFN-γ and/or TNF-α or by the percentage of T cells with CD4 or CD8 phenotype that expressed CD107a (CD4 or CD8 CTLs).

As shown in Figure 4B after three consecutive intranodal immunizations of RNA-TMEP, total HIV-1 TMEP-specific (Gag + GPN) CD4 and to a lesser extent CD8 T cell responses were observed in inguinal LNs (upper panel). Regarding CD107a expression as an indirect marker of cytotoxic activity (lower panel), intranodal immunizations of RNA-TMEP also induced TMEP-specific CD8 CTLs in inguinal LNs.

After intramuscular MVA boost (Figure 4C), the HIV-1-specific T cell responses were potently enhanced and mainly mediated by the CD8 T cell compartment. DNA-TMEP/MVA-TMEP immunization regimen induced the highest TMEP-specific CD8 T cell responses (*p* < 0.001), followed by the heterologous combination RNA-TMEP/MVA-TMEP both in spleen and inguinal LNs (upper panels). Regarding CD107a expression (lower panels), TMEP-specific CTL responses were mainly mediated by the CD8 T cells, with DNA-TMEP/MVA-TMEP and RNA-TMEP/MVA-TMEP immunization regimens inducing the highest TMEP-specific CD8 CTL responses in both organs.

Finally, we also evaluated the vector VACV E3-specific CD8 T cell response elicited after MVA-WT or MVA-TMEP boost immunization. As shown in Figure 4D, the vector E3-specific CD8 T cell responses were mainly detected in the spleen from immunized animals. PBS-primed groups (PBS/MVA-WT and PBS/MVA-TMEP) induced higher levels of E3-specific CD8 T cells than RNA- or DNA-primed groups (RNA-TMEP/MVA-TMEP and DNA-TMEP/MVA-TMEP), suggesting an inverse correlation between HIV-1- and VACV-specific T cell responses.

### 3.6. Priming with Unmodified RNA-TMEP and Booster with MVA-TMEP Triggered Higher and Longer-Term CD8^+^ T Cell Responses than the Modified RNA-TMEPmod Vector

**Figure 5:**
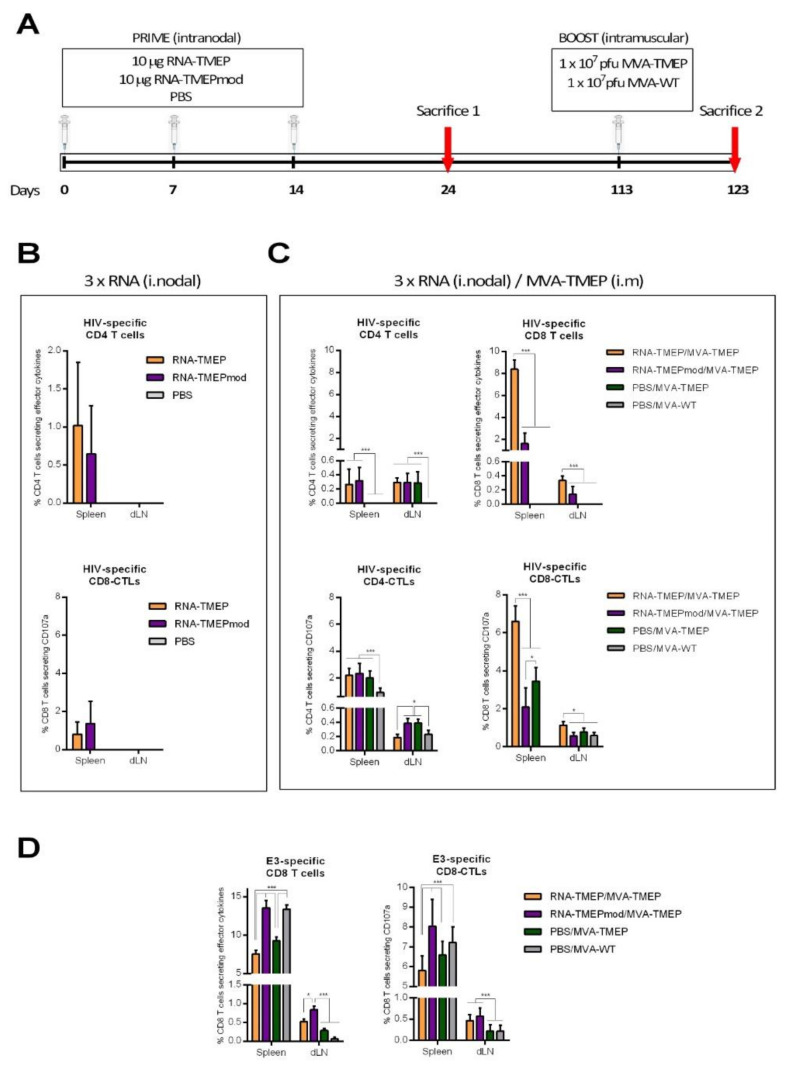
Effect of the modification of RNA-TMEP vector with 1-methyl-3′-pseudouridylyl (RNA-TMEPmod) on the T cell response induced in BALB/c mice after intranodal RNA-TMEP or RNA-TMEPmod prime followed by a late intramuscular MVA-TMEP boost. (**A**) Immunization schedule. (**B**) Magnitude of the total TMEP-specific CD4 T cell responses measured after the third intranodal immunization by ICS assay following stimulation of splenocytes or lymphocytes from inguinal LNs with HIV-1 clade B consensus peptide pools. The total value in each group represents the sum of the percentages of CD4^+^ T cells secreting IL-2 and/or IFN-γ and/or TNF-αeffector cytokines (upper panel) or the percentages of CD8^+^ T cells expressing CD107a against HIV-1 clade B consensus peptide pools (lower panel). (**C**) Magnitude of the TMEP-specific CD4 and CD8 T cell responses measured after a late intramuscular MVA boost by ICS assay following stimulation of splenocytes or lymphocytes from inguinal LNs with HIV-1 clade B consensus peptide pools. The total value in each group represents the sum of the percentages of CD4^+^ or CD8^+^ T cells secreting IL-2 and/or IFN-γ and/or TNF-αeffector cytokines (upper panels) or the percentages of CD4^+^ or CD8^+^ T cells expressing CD107a (lower panels) against HIV-1 clade B consensus peptide pools. (**D**) Magnitude of the VACV E3-specific CD8 T cell responses measured after intramuscular MVA boost by ICS assay following stimulation of splenocytes or lymphocytes from inguinal LNs with VACV E3 peptide. The total value in each group represents the sum of the percentages of CD8^+^ T cells secreting IL-2 and/or IFN-γ and/or TNF-αeffector cytokines (left panel) or the percentages of CD8^+^ T cells expressing CD107a (right panel) against E3 peptide. All data are background subtracted. 95% CI is represented. * *p* < 0.05; *** *p* < 0.001.

Next, we analyzed the potential effect of the introduction of the 1-methyl-3′-pseudouridylyl modification in RNA-TMEP on the immunogenicity elicited by this vector. For this, groups of BALB/c mice (*n* = 4) were immunized as described in the Materials and Methods section. At 10 days post late MVA boost (day 123), the animals were sacrificed and their spleens and inguinal LNs processed for ICS assay to determine both HIV-1- and VACV-specific T cell responses (Figure 5A). The stimulation of the cells and the analysis of the HIV-1- or VACV-specific T cells were performed as described above.

As shown in Figure 5B, after three consecutive intranodal immunizations of RNA-TMEP or RNA-TMEPmod, total HIV-1 TMEP-specific CD4 T cell responses (upper panel) were observed in spleens from animals immunized with RNA-TMEP or RNA-TMEPmod, although no differences between both mRNA constructs were detected. Regarding CD107a expression (lower panel), intranodal immunizations of RNA-TMEP or RNA-TMEPmod induced TMEP-specific CD8 CTLs in the spleen but again no differences between the mRNAs were observed.

After a late intramuscular MVA-TMEP boost (Figure 5C), the HIV-1-specific T cell responses were potently enhanced and, as previously observed, were mainly mediated by the CD8 T cell compartment. RNA-primed groups (RNA-TMEP/MVA-TMEP and RNA-TMEPmod/MVA-TMEP) were the only groups that induced TMEP-specific CD8 T cells both in spleen and inguinal LNs (upper panels). The RNA-TMEP-primed group elicited higher CD8^+^ T cells than the RNA-TMEPmod-primed group (*p* < 0.001). Regarding CD107a expression (lower panels), TMEP-specific CD4 and CD8 CTLs were detected both in spleens and inguinal LNs, with RNA-TMEP/MVA-TMEP immunization regimen inducing the highest TMEP-specific CD8 CTL responses in both organs, suggesting that the modification introduced in RNA-TMEP vector negatively affects its immunogenicity.

Vector VACV E3-specific CD8 T cell responses elicited after MVA-WT or MVA-TMEP boost were mainly observed in the spleens from immunized animals. As shown in Figure 5D, the PBS/MVA-WT and RNA-TMEPmod/MVA-TMEP groups induced the highest E3-specific CD8 T cells, suggesting again an inverse correlation between HIV-1- and VACV-specific T cell immune responses.

## 4. Discussion

While there has been a major effort in the development of a vaccine against HIV infection, this goal has not been accomplished with success and novel strategies are being sought. The recent pandemic caused by the coronavirus SARS-CoV-2 is focusing the vaccine field in the direction of mRNA molecules as the new platform of vaccines against infectious pathogens. This is indeed supported by the successes of the Pfizer and Moderna vaccines, both containing mRNA encoding the membrane-bound full-length stabilized pre-fusion S (spike) protein of SARS-CoV-2 encapsulated in a lipid nanoparticle. Phase III clinical trials with both vaccines have shown efficacies above 90% against SARS-CoV-2 infection, and currently hundreds of millions of people are being vaccinated with these mRNA vaccines [7,8].

Undoubtedly, the great success of the mRNA vaccines against SARS-CoV-2 will be translated into new mRNA vaccines in the HIV field. With this in mind, in this investigation we have generated two types of mRNA vectors expressing a multiepitopic HIV-1 protein specially designed to contain T cell protective conserved epitopes from HIV-1 Gag, Pol and Nef proteins [2]. One of the HIV-1 mRNA vectors was modified by the incorporation of 1-methyl-3′-pseudouridylyl in RNA-TMEP and the other was unmodified. The nucleoside modification in the mRNA was added to enhance the mRNA stability and to avoid the innate immune response mounted by the host [9].

We wanted to establish: (i) the potential differences in expression levels and innate immune responses elicited between both mRNA vectors; (ii) the immunological profile induced in an animal model after mRNA delivery by the intranodal route; and (iii) the immunological benefit of the prime/boost administration in mice of the mRNA combined with the poxvirus vector MVA expressing the same antigen.

We showed that the expression levels of unmodified RNA-TMEP in cultured cells of different origins are higher than the levels observed with the modified mRNA, with both vectors triggering distinct innate immune signatures. When administered by the intranodal route in mice, the unmodified mRNA activated low levels of HIV-1-specific CD4 and CD8 T cells and this effect was markedly increased when animals received a booster dose with the MVA-TMEP vector expressing the same multiepitopic protein. The differences may be determined by differential stability within the transfected cells of the two mRNA molecules, probably mediated by the type of innate immune responses induced by each mRNA.

A head-to-head comparison between both mRNAs revealed higher T cell stimulation after MVA booster with the unmodified mRNA. This enhancement could be related to the higher uptake levels in cells by unmodified mRNA as well as to the differential induction of immunomodulatory molecules. In fact, unmodified mRNA triggered higher levels of RANTES, IFIT1, IL1β, IL10 and IL12 than modified mRNA in the macrophage cell line THP-1, while the modified mRNA enhanced the expression of IFIT2, IFNβ, RIG-I and IL6 compared to unmodified mRNA. Clearly, the combination of an mRNA and a MVA vector expressing the same antigen is a powerful approach to enhance the HIV-1-specfic T cell immune responses. Moreover, the immune enhancement was also observed after three and a half months of the 3 priming doses, indicating activation of T cell memory responses.

We have selected the intranodal route for mRNA administration since we wanted to determine the direct effect of naked mRNA in this highly immune-activating tissue. In fact, the intranodal route of mRNA administration has been used in patients vaccinated against tumors with mRNA encoding neoantigens [10,11,12] and this approach is experiencing a major boost as a vaccination regimen. Intranodal delivery of ‘naked’ mRNA has been reported to induce both higher protein expression and protective antigen-specific T cell responses compared to subcutaneous and intradermal injections [13]. Then, the efficacy of mRNA administered into lymph nodes depends on its uptake and ability to generate a CTL-inducing milieu. In this sense, our results on human dendritic cells maturation suggested that mRNA administration could enhance the stimulatory capacity of lymph node-resident dendritic cells, as it was observed in dendritic cell electroporation and lipofection assays. However, it is not clear whether the intrinsic adjuvant effect of mRNA is sufficient to fully exploit the immunostimulatory capacity of dendritic cells or whether additional stimulation signals are required [14]. In the HIV field, previous published results have shown that uptake of mRNA encoding strong activation signals and a potent HIV-1 antigen confers to dendritic cells a T cell stimulatory capacity and enhances their ability to stimulate antigen-specific immunity both in vitro and in vivo [15]. In this context, the important biological issue highlighted here is that both naked mRNAs trigger HIV-1-specific immune responses when delivered by the intranodal route.

Recently, the Oxford group presented the findings in mice of a combination of self-amplifying mRNA vaccines with the AstraZeneca (adenovirus) vaccine, evidencing that prime/boost combination was a superior protocol for activation of SARS-CoV-2-specific immune responses than the homologous vectors, in terms of antibody levels (total and neutralizing) as well as activation of T cell responses [16]. In fact, recruitment for phase I/II clinical trials with the AstraZeneca and Pfizer mRNA SARS-CoV-2 vaccine combination is currently ongoing. Clinical trials with individuals that received the first dose from AstraZeneca and the second dose of Pfizer mRNA vaccine revealed potentiation of the immune responses, as measured by increased S protein binding antibodies and enhanced neutralizing antibody levels [17,18]. Thus, heterologous combinations of vaccines might provide an advantage for vaccination.

In this regard, the first demonstration of the benefit of combined vaccines, and the order of immunization to enhance immunogenicity and protection against a pathogen, was shown in the malaria model with the combination of two vectors: influenza and a poxvirus expressing the malaria CS (circumsporozoite) antigen [19,20,21].

Overall, our results showed the use of an mRNA vaccine combined with the poxvirus vector MVA as a promising protocol of vaccination against HIV-1. The combined vaccination triggered specific immune responses in mice that might be relevant in protection. This potential benefit should be demonstrated in non-human primates and in clinical trials. An intranodal phase I/II therapeutic intervention with mRNAs in HIV-infected patients as part of a EU-funded HIVACAR program is planned for 2022.

## 5. Conclusions

We have generated two types of mRNA vectors expressing a multiepitopic HIV-1 protein containing T cell protective conserved epitopes from HIV-1 Gag, Pol and Nef proteins. One of the HIV-1 mRNA vectors was modified by the incorporation of 1-methyl-3′-pseudouridylyl in RNA (RNA-TMEPmod) and the other was unmodified (RNA-TMEP). The expression levels of unmodified RNA-TMEP in cultured cells of different origins are higher than the levels observed with the modified mRNA and both RNA vectors triggered a differential induction of innate immune molecules. When administered by the intranodal route in mice, the unmodified mRNA activated low levels of HIV-1-specific CD4 and CD8 T cells and this activation was potently enhanced after a booster dose with the MVA-TMEP vector expressing the same multiepitopic antigen. These results showed the combined use of an mRNA vaccine with the poxvirus vector MVA as a promising vaccination regimen against HIV-1 that might be applicable to human vaccination programs.
